# Finite-Size Thermodynamics of the Two-Dimensional Dipolar *Q*-Clock Model

**DOI:** 10.3390/e28020181

**Published:** 2026-02-05

**Authors:** Michel Aguilera, Francisco J. Peña, Eugenio E. Vogel, Patricio Vargas

**Affiliations:** 1 Instituto de Física, Pontificia Universidad Católica de Valparaíso, Casilla 4950, Valparaíso 2373223, Chile; 2 Departamento de Física, Universidad Técnica Federico Santa María, Av. España 1680, Valparaíso 2390123, Chile; patricio.vargas@usm.cl; 3 Departamento de Ciencias Físicas, Universidad de La Frontera, Casilla 54-D, Temuco 4811230, Chile; eugenio.vogel@ufrontera.cl; 4 Facultad de Ingeniería y Arquitectura, Universidad Central de Chile, Av. Sta. Isabel 1186, Santiago 8330601, Chile

**Keywords:** Q-clock model, dipolar interactions, ground-state multiplicity, thermodynamics

## Abstract

We present a fully controlled thermodynamic study of the two-dimensional dipolar *Q*-state clock model on small square lattices with free boundaries, combining exhaustive state enumeration with noise-free evaluation of canonical observables. We resolve the complete energy spectra and degeneracies $\left\{E_{n},c_{n}\right\}$ for the Ising case (Q=2) on lattices of size L=3,4,5, and for clock symmetries Q=4,6,8 on a 3×3 lattice, tracking how the competition between exchange and long-range dipolar interactions reorganizes the low-energy manifold as the ratio α=D/J is varied. Beyond a finite-size characterization, we identify several qualitatively new thermodynamic signatures induced solely by dipolar anisotropy. First, we demonstrate that ground-state level crossings generated by long-range interactions appear as exact zeros of the specific heat in the limit C(T→0,α), establishing an unambiguous correspondence between microscopic spectral rearrangements and macroscopic caloric response. Second, we show that the shape of the associated Schottky-like anomalies encodes detailed information about the degeneracy structure of the competing low-energy states: odd lattices (L=3,5) display strongly asymmetric peaks due to unbalanced multiplicities, whereas the even lattice (L=4) exhibits three critical values of α accompanied by nearly symmetric anomalies, reflecting paired degeneracies and revealing lattice parity as a key organizing principle. Third, we uncover a symmetry-driven crossover with increasing *Q*: while the Q=2 and Q=4 models retain sharp dipolar-induced critical points and pronounced low-temperature structure, for Q≥6, the energy landscape becomes sufficiently smooth to suppress ground-state crossings altogether, yielding purely thermal specific-heat maxima. Altogether, our results provide a unified, size- and symmetry-resolved picture of how long-range anisotropy, lattice parity, and discrete rotational symmetry shape the thermodynamics of mesoscopic magnetic systems. We show that dipolar interactions alone are sufficient to generate nontrivial critical-like caloric behavior in clusters as small as 3×3, establishing exact finite-size benchmarks directly relevant for van der Waals nanomagnets, artificial spin-ice arrays, and dipolar-coupled nanomagnetic structures.

## 1. Introduction

Low-dimensional magnetic systems with long-range interactions provide a fertile setting for exploring the interplay between geometry, anisotropy, and cooperative effects in mesoscopic materials. Among the paradigmatic models used to describe such systems, the *Q*-state clock model has emerged as a minimal yet versatile framework for two-dimensional spins with discrete rotational symmetry, interpolating smoothly between the Ising (Q=2) [1,2] and XY [3,4,5,6,7] (Q→∞) limits. Even in the absence of long-range couplings, its thermodynamic behavior is remarkably rich, exhibiting discrete-symmetry ordering for all *Q* and Berezinskii–Kosterlitz–Thouless (BKT) physics for Q≥4 [8].

The inclusion of dipolar interactions adds a further layer of complexity. Dipolar couplings decay slowly ($\propto r^{-3}$), placing them within the broader class of long-range interactions characterized by nontrivial critical behavior [9,10,11]. Their strong anisotropy promotes frustrated magnetic textures and competing domain structures, playing a central role in modern two-dimensional magnets such as van der Waals materials (e.g., CrI_3_, CrGeTe_3_, Fe_3_GeTe_2_) [12,13], as well as in artificial spin-ice arrays and dipolar-coupled nanomagnets, where stripe phases, domain patterns, and topological defects emerge from the competition between short- and long-range interactions [14,15,16,17].

Despite this relevance, the combined effects of discrete clock symmetry, long-range dipolar anisotropy, and finite cluster geometry remain comparatively unexplored, particularly in small systems where the microscopic energy spectrum can be resolved exactly. Finite clusters are of special interest for two complementary reasons. First, finite-size effects and boundary conditions strongly modify thermodynamic responses: they round critical singularities, shift specific-heat maxima, suppress vortex proliferation, and enhance degeneracy-driven features absent in the thermodynamic limit. Second, for sufficiently small lattices, the full spectrum of the Hamiltonian can be obtained exactly, enabling a noise-free evaluation of thermodynamic observables and a direct correspondence between microscopic level crossings and macroscopic response functions [18,19,20].

An explicit novelty of the present work lies in establishing a direct and degeneracy-resolved correspondence between microscopic spectral reorganizations and macroscopic thermodynamic signatures in dipolar clock models. Specifically, we demonstrate that dipolar interactions alone can induce sharp ground-state level crossings in finite clusters, which manifest as exact zeros of the specific heat in the limit T→0. These zero-temperature anomalies provide a thermodynamic fingerprint of dipolar-driven level rearrangements and allow us to unambiguously relate caloric features to changes in ground-state multiplicity.

Furthermore, by systematically comparing different clock symmetries and lattice sizes, we identify lattice parity as an organizing principle that controls the symmetry or asymmetry of the resulting low-temperature Schottky anomalies. Odd and even lattices display qualitatively distinct caloric responses due to unbalanced versus paired degeneracies of low-energy states. Finally, extending the analysis across increasing clock resolution *Q*, we show that ground-state crossings and degeneracy-driven anomalies are progressively suppressed as the angular discretization approaches the continuous limit, leading to smooth finite-size crossovers. To the best of our knowledge, this combined degeneracy-resolved, parity-sensitive, and *Q*-systematic characterization has not been explicitly established in previous studies of dipolar Ising or *Q*-state clock models.

In this work, we investigate the dipolar *Q*-state clock model on small square lattices with free boundaries, combining exact enumeration of all spin configurations with analytical evaluation of canonical thermodynamic observables. We resolve the full sets of energies and degeneracies $\left\{E_{n},c_{n}\right\}$ for clock symmetries Q=2,4,6,8 on a 3×3 lattice, as well as for the Ising case (Q=2) on lattices of size L=3,4,5. By varying the dipolar ratio α=D/J, we track how the competition between exchange and dipolar interactions reshapes the low-energy spectrum and manifests in the specific heat, entropy, and ground-state multiplicity. This controlled finite-size setting allows us to identify robust signatures of dipolar-induced spectral reorganizations across lattice sizes and discrete symmetries.

Our analysis reveals several key phenomena. For Q=2 and Q=4, the system exhibits ground-state crossings at well-defined values of α, which appear as zeros of the specific heat in the limit C(T→0,α). The Schottky-like anomalies that emerge around these points encode the degeneracy structure of the low-lying spectrum, with odd lattices (L=3,5) displaying strongly asymmetric peaks due to unbalanced multiplicities, and the even lattice (L=4) showing nearly symmetric anomalies associated with paired degeneracies. In contrast, for Q≥6 the energy landscape becomes progressively smoother, ground-state crossings disappear within the explored range of α, and the specific heat exhibits only broad, purely thermal maxima, reflecting the increasing angular continuity of the spin space.

Beyond the strictly low-temperature regime, the Ising model (Q=2) develops a double-peak structure in the specific heat over a finite interval of α, signaling the coexistence of bulk-like thermal excitations and additional low-energy modes generated by the competition between exchange and dipolar interactions. For higher clock symmetries (Q≥6), increasing α suppresses this double-peak structure as the dipolar interaction reorganizes the low-energy spectrum and drives the ground state away from an exchange-stabilized ferromagnetic configuration toward textures governed primarily by long-range anisotropy.

Although the finite-size systems considered here do not host a genuine Berezinskii–Kosterlitz–Thouless phase transition, the caloric features observed for Q≥6 bear a qualitative resemblance to the crossover phenomena associated with vortex–antivortex physics in the two-dimensional XY model [4,5]. In this sense, dipolar interactions effectively smooth the discrete angular structure of the clock model, promoting gradual thermal activation rather than sharp degeneracy-driven anomalies.

Taken together, these results demonstrate that dipolar interactions alone are sufficient to generate nontrivial caloric behavior, including critical-like features associated with ground-state level crossings, in clusters as small as 3×3. The present study therefore provides a controlled finite-size benchmark for interpreting experiments on van der Waals nanomagnets, patterned dipolar arrays, and mesoscopic magnetic textures, where discrete symmetry, long-range anisotropy, and cluster geometry jointly determine the thermodynamic response.

The remainder of this article is organized as follows. Section 2 introduces the Hamiltonian, discusses the role of dipolar anisotropy, and outlines the exact enumeration approach. Section 3 presents the thermodynamic properties of the model, including internal energy, entropy, specific heat, ground-state multiplicity, and the effects of lattice size, parity, and clock symmetry. Section 4 discusses the experimental relevance and feasibility of the explored parameter regime. Section 5 summarizes the main findings and discusses perspectives for extending this exact approach to larger clusters and dipolar-coupled nanomagnetic systems.

## 2. Model and Methods

### 2.1. General Definitions

**Figure 1:**
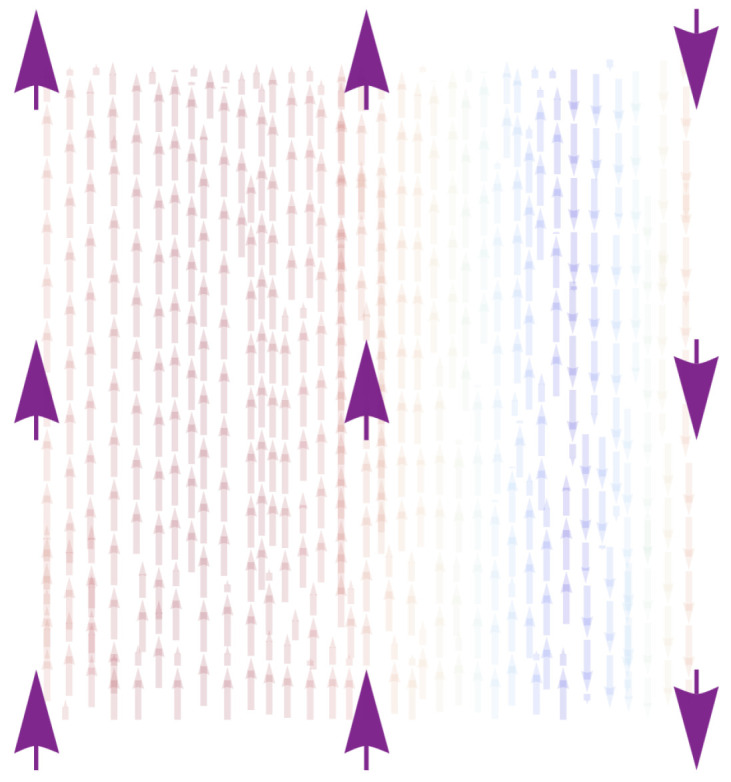
Schematic 2D configuration (Ising limit Q=2) on a square lattice with free boundaries for L=3. Arrows denote the Ising spin orientation (↑ or ↓). The color shading is used only to visually distinguish regions with different spin alignment and does not represent an additional thermodynamic variable.

We consider the two-dimensional *Q*-state clock model on a square lattice of size L×L (number of sites $N=L^{2}$) with free boundaries, where each local magnetic moment (“spin”) $S_{i}$ at site *i* points along one of *Q* equally spaced directions in the plane. Writing $S_{i}=\left(\sin\theta_{i},\cos\theta_{i}\right)$ with $\theta_{i}=2\pi k/Q$, k=0,1,…,Q−1, the model provides a natural interpolation between the Ising limit (Q=2) and the XY model (Q→∞). Throughout, spins are dimensionless unit vectors, and we set $k_{B}=1$. The working Hamiltonian includes nearest-neighbor exchange, an in-plane Zeeman field, and long-range dipolar couplings,

(1) $$H=-J\sum_{\langle i,j\rangle }S_{i}\cdot S_{j}-B\sum_{i}n_{B}\cdot S_{i}+D\sum_{i>j}\frac{S_{i}\cdot S_{j}-3\left(S_{i}\cdot n_{r}\right)\left(S_{j}\cdot n_{r}\right)}{r_{ij}^{3}},$$

which can be rewritten as:

(2) $$H\;=\;-J\sum_{\langle i,j\rangle }\cos\left(\theta_{i}-\theta_{j}\right)\;-\;B\sum_{i}\cos\theta_{i}\;-\frac{D}{2}\;\sum_{i>j}\frac{\cos\left(\theta_{i}-\theta_{j}\right)+3\cos\left(\theta_{i}+\theta_{j}-2\phi_{ij}\right)}{r_{ij}^{3}}.$$

where J>0 is the ferromagnetic exchange between nearest neighbors 〈i,j〉, *B* is an external field (chosen along the *y*-axis, $n_{B}=\left\{0,1\right\}$), *D* sets the dipolar strength, $r_{ij}=\left|r_{i}-r_{j}\right|$, and $\phi_{ij}$ is the polar angle of the vector $r_{ij}$ in the lattice plane (unit vector $n_{r}$). Unless stated otherwise, energies and temperatures are expressed in units of *J*, and we use the dimensionless ratio α=D/J to quantify the relative dipolar coupling (Figure 1).

Long-range dipolar terms frustrate local alignment and reshape the energy landscape, competing with exchange and field contributions. In two dimensions, this competition intertwines with topology: for Q≥4 the clock model supports a Berezinskii–Kosterlitz–Thouless (BKT) regime associated with vortex–antivortex physics. In contrast, the underlying discrete *Q*-fold rotational symmetry ($Z_{Q}$) of the clock variables leads to an additional discrete-symmetry ordering at lower temperatures [8].

Finite geometry and boundary conditions play a decisive role in the present context. In small lattices, where the linear system size *L* (defined as the number of spins per lattice edge) becomes comparable to the correlation length ξ(T) measured in lattice units, cooperative fluctuations are strongly suppressed by finite-size effects. In this mesoscopic regime, specific-heat maxima are rounded and shifted, and the proliferation of collective or topological excitations is impeded, as correlations cannot fully develop across the system. As a result, intuition based on the thermodynamic limit is not directly transferable to finite samples. Free boundaries further enhance edge contributions and modify bulk-like thermodynamic signatures relative to periodic boundary conditions.

Throughout this work, we therefore consider finite clusters with free boundary conditions. This choice is physically motivated by mesoscopic realizations such as artificial spin-ice arrays, magnetic nanoislands, or confined van der Waals magnets, where edges are intrinsic and experimentally relevant rather than a finite-size artifact.

While periodic or antiperiodic boundary conditions can be straightforwardly implemented for the short-range exchange interaction, the situation is fundamentally different for the long-range dipolar term. A mathematically controlled implementation of periodic boundaries for dipolar $1/r^{3}$ interactions requires including interactions with all periodic images of the simulation cell, typically via Ewald-type lattice summations or equivalent image-sum techniques. Naive truncated or minimum-image implementations would instead define a different Hamiltonian and introduce uncontrolled cutoff- and geometry-dependent effects, rather than constituting a genuine change of boundary conditions.

Since the focus of the present study is on exact finite-cluster thermodynamics, we consistently adopt free boundary conditions for the dipolar interaction. A fully periodic treatment of dipolar sums, while certainly of interest, lies beyond the scope of the present work and is left for future investigation.

### 2.2. Computational Strategy and Observables

To capture thermodynamics across different sizes and symmetries, we perform exact spin-configuration enumeration on small lattices. The calculation of thermal averages, a classic problem in statistical mechanics, has been approached through a spectrum of methods: transfer-matrix solutions in one and two dimensions [21,22,23,24], mean-field theory [25,26], the Metropolis algorithm in Monte Carlo simulations [27,28,29], and more recently, through exotic statistical frameworks and diverse machine-learning–based selection techniques [30,31,32,33]. Without loss of generality, we explore exact state counting in order to lay the groundwork for implementing long-range interactions in simulations of this kind.

For different clock models on a 3×3 lattice and for the Ising model with L=3,4,5, the complete energy spectrum $\left\{E_{n}\right\}$ and corresponding degeneracies $\left\{c_{n}\right\}$ are obtained through exhaustive enumeration of all $Q^{N}$ spin configurations. By construction, the degeneracies satisfy the closure relation $\sum_{n}c_{n}=Q^{N}$, ensuring that the full configuration space is accounted for. The energetic levels $E_{n}$ depend explicitly on the Hamiltonian parameters {J,B,D}; throughout this work, their relative influence is conveniently characterized by the dimensionless dipolar ratio α=D/J. This allows us to construct the canonical partition function

(3) $$Z\left(T,\left\{J,B,D\right\};Q,L\right)=\sum_{n}c_{n}\;e^{-E_{n}\left(\left\{J,B,D\right\}\right)/T}.$$

The complete set of configurations explored, determined by clock symmetry and lattice size, is summarized in Table 1. Here, *T* denotes the temperature of the thermal bath, while the energetic states incorporate exchange, Zeeman, and dipolar couplings. Thermodynamic observables then follow exactly:

(4) $$U\left(T,\alpha \right)=-\frac{\partial }{\partial \beta }\ln Z,\;C\left(T,\alpha \right)=\frac{\partial U}{\partial T},\;S\left(T,\alpha \right)=\frac{U-F}{T},$$

with

(5) F(T,α)=−TlnZ.

and, when relevant, the magnetization $M\left(T,\alpha \right)=-\frac{\partial F}{\partial B}$. This enumeration provides noise-free benchmarks for the effects of α, *Q*, and *L*, and resolves how each Hamiltonian term contributes to the thermodynamic density of states.

When assessing BKT-like behavior for Q≥4, we track finite-size trends of response functions and, where informative, stiffness-related proxies consistent with the discrete symmetry. Across all calculations, we explore representative sets in the (J,D) plane at fixed *B*, reporting results as functions of α for Ising and for Q=4,6,8 and size *L*. This mixed exact-numerical protocol isolates finite-size from interaction-induced effects in a controlled manner, enabling a coherent thermodynamic description of the dipolar clock model from the microscopic, enumeration-accessible regime to mesoscopic lattices where collective behavior and long-range couplings jointly dictate the observed caloric and magnetic responses.

### 2.3. Remark on Computational Feasibility and Model Truncations

The exponential growth of the configuration space with both *L* and *Q* is illustrated in Table 1. Even for moderate sizes, the number of spin configurations quickly exceeds any realistic enumeration capacity, reaching $10^{32}$–$10^{44}$ states for L≥6 and Q≥8. This scaling motivates the transition from exact enumeration to Monte Carlo sampling. For dipolar interactions, further care must be taken: a naive truncation of the long-range $1/r^{3}$ potential introduces nonphysical artifacts in the thermodynamic limit [34,35]. For our study, we will limit ourselves to $10^{8}$ configurations.

### 2.4. Partition Function for Small Systems

We first analyze the thermodynamics of finite lattices by explicitly enumerating microstates. For a given lattice size L×L ($N=L^{2}$), clock symmetry *Q*, external field *B* (contributing to the lifting of ground-state degeneracies), and dipolar ratio α=D/J (for simplicity J=1 and α contains the dipolar information), the canonical partition function reads

(6) $$Z\left(T,\alpha ;B,Q,L\right)\;=\;\sum_{n=1}^{\lambda }c_{n}\;e^{-E_{n}/T},$$

where ${\left\{E_{n}\right\}}_{n=1}^{\lambda }$ are the distinct energy levels of the Hamiltonian in Equation (2) and $c_{n}$ their corresponding degeneracies (number of spin configurations yielding $E_{n}$). Exact enumeration provides $\left\{E_{n},c_{n}\right\}$ without statistical error and thus a noise-free benchmark for all thermodynamic observables derived from *Z*. To gain intuition on how each Hamiltonian contribution shapes the spectrum at small sizes, we also resolve the energy into exchange, Zeeman, and dipolar parts for every configuration,

(7) $$\begin{array}{cc} E & =E^{\left(J\right)}+E^{\left(B\right)}+E^{\left(D\right)},\\ E^{\left(J\right)} & =-J\;\sum_{\langle i,j\rangle }\;\cos\left(\theta_{i}-\theta_{j}\right),\\ E^{\left(B\right)} & =-B\;\sum_{i}\cos\theta_{i},\\ E^{\left(D\right)} & =-\frac{D}{2}\;\sum_{i>j}\frac{\cos\left(\theta_{i}-\theta_{j}\right)+3\cos\left(\theta_{i}+\theta_{j}-2\phi_{ij}\right)}{r_{ij}^{3}}. \end{array}$$

From the enumerated set we construct discrete histograms (degeneracy densities) $G^{\left(\nu \right)}\left(E\right)=\sum_{\mathrm{configs}}\delta_{E,E^{\left(\nu \right)}}$ for ν∈{J,B,D}, which compactly encode how each term contributes to the distribution of energies in a finite sample (fixed thermal bath for enumeration).

### 2.5. Energy Histograms for Q=2 and L=3

**Figure 2:**
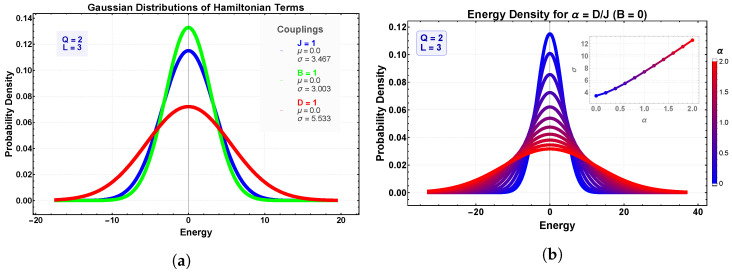
Gaussian modeling of energy spectra for the Ising case (Q=2) on a 3×3 lattice. (**a**) Individual contributions from exchange, Zeeman, and dipolar energies. (**b**) Broadening and reshaping of the total spectrum as the dipolar ratio α=D/J increases. The parameter σ denotes the standard deviation (energy width) of the Gaussian fits to the corresponding histograms, providing a quantitative measure of the dispersion of energy levels. Its increase with α reflects the enhanced energetic heterogeneity induced by long-range dipolar interactions in finite clusters.

As a concrete reference, Figure 2a displays the histograms of $E^{\left(J\right)}$, $E^{\left(B\right)}$ (with B=1 in units of *J*), and $E^{\left(D\right)}$ for the Ising limit Q=2 on a 3×3 lattice with free boundaries [36]. The Zeeman contribution exhibits the narrowest spread, consistent with an effectively noninteracting alignment cost relative to the field direction. The exchange term shows a broader distribution reflecting the combinatorial variety of nearest-neighbor bonds. The dipolar contribution is the broadest, owing to its long-range character and angular dependence through $3\cos(\theta_{i}+\theta_{j}-2\phi_{ij})$, which significantly increases the spread of pairwise energies across the lattice.

### 2.6. Dependence on the Dipolar Ratio α=D/J

Figure 2b shows how the spectrum evolves as α is varied for the same Q=2, L=3 system. Increasing α broadens and skews the dipolar distribution more strongly than the exchange or Zeeman terms, causing the total energy spectrum $\left\{E_{n}\right\}$ to spread accordingly. At large α, finite-size discretization of $\left\{r_{ij},\phi_{ij}\right\}$ under free boundaries generates small multi-peak structures intrinsic to mesoscopic clusters but absent in the thermodynamic limit.

### 2.7. Dependence on the Clock Symmetry *Q*

**Figure 3:**
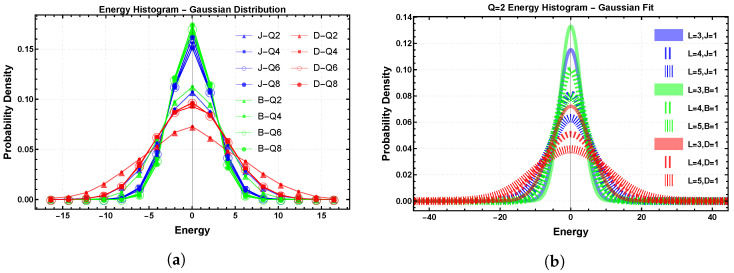
Gaussian modeling of energy spectra of the dipolar clock model. (**a**) Histograms for L=3 and Q=2,4,6,8: increasing *Q* enriches the number of accessible energy levels and smooths the spectrum. (**b**) Histograms for Q=2 and L=3,4,5: increasing lattice size adds distinct energy levels and modifies degeneracy patterns; free boundaries enhance edge effects.

Increasing the number of angles from Q=2 to Q=4,6,8 enriches the set of local orientations $\left\{\theta_{i}\right\}$ and therefore the multiplicity of distinct pair energies, i.e., the interaction energies associated with distinct spin pairs (i,j) for given angular configurations $\left(\theta_{i},\theta_{j}\right)$. Figure 3a compares histograms at fixed L=3 for several *Q*. As *Q* increases, the exchange term interpolates toward a quasi-continuous XY-like distribution, and the Zeeman term develops intermediate values between extreme alignments. The dipolar contribution remains the most dispersive and the most sensitive to increasing *Q*, reflecting the interplay between angular anisotropy and long-range coupling.

### 2.8. Finite-Size Effects at Fixed *Q*

Figure 3b shows how the histograms evolve with system size for the Ising case Q=2, comparing L=3,4,5. Increasing *L* increments the number of distinct levels and changes their degeneracy patterns. Free boundaries accentuate edge-dominated splittings, which play a key role in understanding finite-size effects on the rounding and shifting of thermodynamic anomalies such as the specific-heat peaks.

The enumerated sets $\left\{E_{n},c_{n}\right\}$ obtained in this section directly feed Equation (6), enabling exact evaluation of U(T,α), C(T,α), S(T,α), and M(T,α) in Section 3. These results provide baseline, noise-free thermodynamics for small lattices and establish controlled trends versus α, *Q*, and *L* for the analysis presented below.

## 3. Thermal Averages

### 3.1. Thermodynamic Behavior Under Isolated Interaction Terms

At low temperatures, the Ising model exhibits a ferromagnetic regime driven by the exchange interaction, which favors alignment between nearest neighbors. As temperature increases, thermal fluctuations progressively destroy the ordered state, leading to a paramagnetic phase with vanishing total magnetization. A pure Zeeman term, in contrast, promotes alignment along the external field direction and therefore requires higher temperatures to fully disorder the system. In *Q*-clock models, increasing *Q* enhances the number of accessible spin orientations and allows for vortex-like excitations.

**Figure 4:**
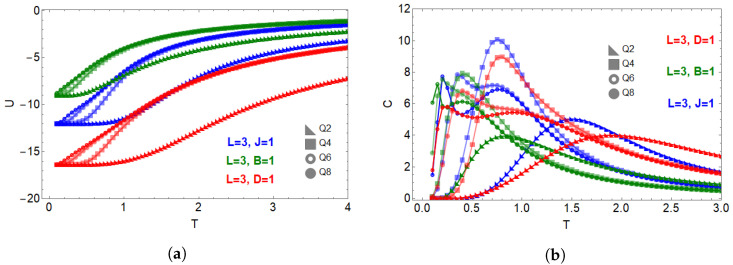
Thermodynamic behavior under isolated interaction terms on an L=3 lattice for Q=2,4,6,8. (**a**) Internal energy U(T) for pure exchange (J=1, blue), Zeeman (B=1, green), and dipolar (D=1, red). (**b**) Specific heat C(T) for the same cases. Exchange interactions display the expected double-peak structure at large *Q*; Zeeman fields suppress the low-temperature peak; dipolar couplings broaden and shift the spectral response.

Figure 4 shows the temperature dependence of the internal energy and specific heat when each interaction term is activated separately on an L=3 lattice. Panel (a) displays U(T): the Zeeman term yields the highest internal energy for all *Q* values, while the exchange interaction produces the lowest energies due to cooperative alignment. The dipolar term stabilizes low-energy configurations, reflecting the strong ordering tendency induced by long-range couplings.

Panel (b) presents the specific heat C(T). For pure exchange (J=1), the cases Q=6 and Q=8 display a characteristic double-peak structure, associated with a BKT-like crossover followed by discrete $Z_{Q}$ ordering in finite systems. The Zeeman term (B=1) suppresses the low-temperature peak, merging it with the main response into a single dominant feature. Dipolar couplings (D=1) broaden the thermal response and shift spectral weight toward higher temperatures, partially merging the two-peak structure for Q≥6.

**Figure 5:**
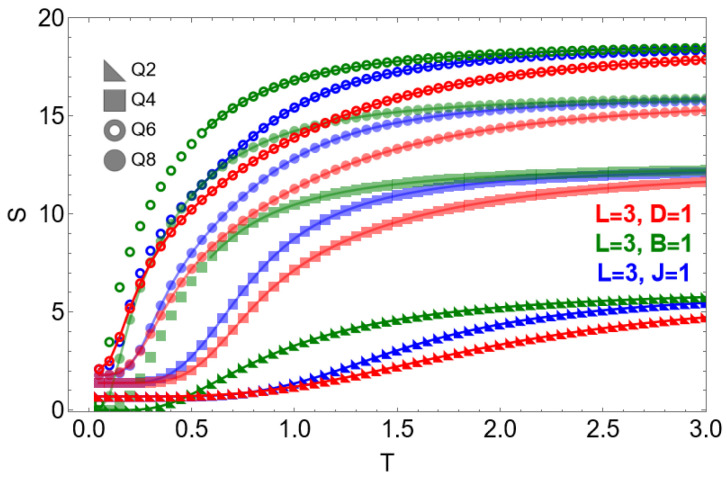
Entropy S(T) for an L=3 lattice under isolated interaction terms: exchange (J=1, blue), Zeeman (B=1, green), and dipolar (D=1, red). Markers indicate clock symmetries Q=2,4,6,8.

Figure 5 shows the entropy S(T) for the same system under isolated interactions. In the pure exchange case, the system approaches a finite residual entropy $S_{0}=\ln\left(Q\right)$ as T→0, reflecting the ground-state degeneracy inherent to the *Q*-clock symmetry. When the Zeeman term is present, this degeneracy is lifted and S→0 as T→0. For dipolar interactions, the system retains a finite residual entropy, as long-range couplings favor frustrated configurations rather than a single ferromagnetic ground state.

At high temperatures, the entropy approaches the expected limit S=Nln(Q), with N=9, yielding approximate values of 6.2, 12.5, 16.1, and 18.7 for Q=2,4,6, and 8, respectively. This asymptotic regime is reached more slowly in the dipolar case, reflecting the reduced statistical weight of nearly ferromagnetic configurations in the presence of competing long-range interactions.

### 3.2. Finite-Size Effects Under Isolated Interaction Terms

The role of lattice size is examined in terms of the system’s extensivity and edge effects in real space. When thermodynamic quantities such as internal energy, specific heat, and entropy are evaluated per site, one expects them to converge as the number of spins increases, approaching their thermodynamic-limit values. The internal energy per site exhibits distinct behaviors depending on the interaction. In the presence of an external field (B=1), the internal energy remains essentially independent of lattice size, reflecting the uniform alignment imposed by the field. In the exchange and dipolar cases, however, the energy per site decreases with increasing lattice size, with the dipolar interaction yielding the lowest energies due to its long-range, partially antiferromagnetic character.

**Figure 6:**
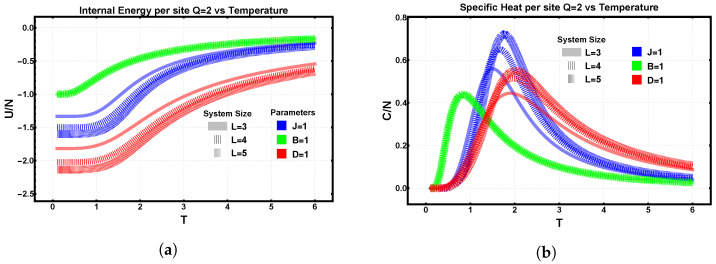
Finite-size effects under isolated interactions in lattices of size L=3,4,5. (**a**) Internal energy per site U/N for exchange (J=1, blue), Zeeman (B=1, green), and dipolar (D=1, red). (**b**) Specific heat per site C/N for the same cases. Exchange and dipolar interactions display sharper peaks whose positions shift toward higher temperatures as *L* increases, reflecting enhanced collective behavior and an approach to thermodynamic-limit trends.

The specific heat per site shows minimal size dependence for the Zeeman interaction but displays a clear trend for the exchange and dipolar terms: the temperature at which the specific heat reaches its maximum increases with system size, signaling enhanced cooperative effects and a shift toward bulk-like behavior. These features are summarized in Figure 6a,b.

**Figure 7:**
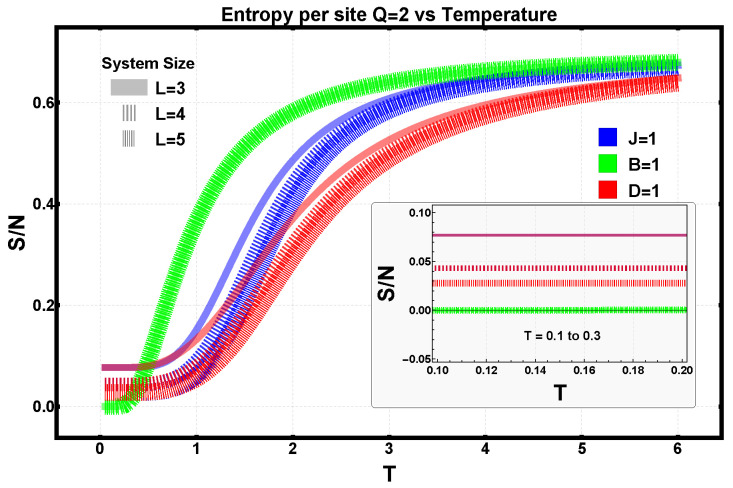
Entropy per site S/N for L=3,4,5 under isolated interaction terms: exchange (J=1, blue), Zeeman (B=1, green), and dipolar (D=1, red). The same color coding applies in the inset. Within each color, the slightly separated curves correspond to different lattice sizes (L=3,4,5), included to explicitly display finite-size effects. This is particularly visible for the dipolar case (red), where the residual entropy depends on lattice size due to frustration. For the Zeeman-only case, the residual entropy vanishes as T→0, consistent with a non-degenerate ground state. At high temperatures, all curves converge to the expected extensive limit.

The entropy per site, shown separately in Figure 7, reveals complementary finite-size trends. In the exchange case, the residual entropy at low temperatures decreases as the lattice grows, consistent with reduced degeneracy and stronger ordering. The Zeeman field drives the entropy to zero as T→0, while the dipolar interaction retains a small but finite residual entropy due to frustration. At very high temperatures, all entropy curves converge to their extensive limits. In the zoom of Figure 7, we note that the degeneracy number of energy-minimizing configurations is the same regardless of whether the Hamiltonian contains an exchange or a dipolar term.

### 3.3. Ising Case: Dependence on the Dipolar Ratio α=D/J

**Figure 8:**
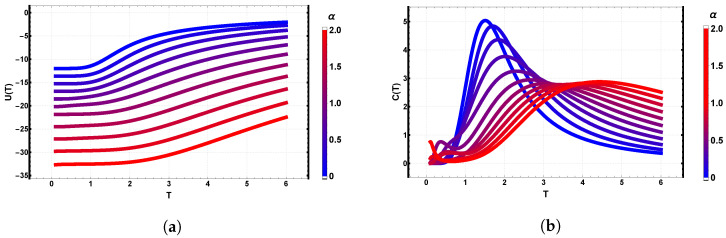
Thermodynamic response of the Ising model (Q=2) on a 3×3 lattice as a function of the dipolar ratio α=D/J. (**a**) Internal energy U(T,α) decreases with α, with the ordering crossover shifting to higher temperatures. (**b**) Specific heat C(T,α) showing a shift of the main peak to higher temperatures and a progressive broadening as α increases; a low-*T* shoulder appears near α≈1.

We now focus on the Ising limit (Q=2) on a 3×3 lattice with B=0, varying the dipolar ratio α∈[0,2] in steps of 0.1. Figure 8a,b summarize the thermal behavior of the internal energy and specific heat. In Figure 8a, the internal energy U(T,α) decreases monotonically with increasing α over the entire temperature range. The inflection point marking the crossover between the low-*T* ordered regime and the high-*T* paramagnetic regime shifts to higher temperatures as α increases, reflecting the strengthening of the dipolar interaction.

Figure 8b shows the corresponding specific heat. As α increases, the main peak broadens and shifts toward higher temperatures, while its height decreases. In an intermediate window around α∼1, a low-*T* shoulder appears, indicative of competing low-energy configurations. For large α, this shoulder merges into a single broad maximum. We observe a tendency towards non-zero specific heat values as T→0 for certain α, revealing a thermal signature of changes in the number of energy-minimizing configurations in the ground state of the system.

**Figure 9:**
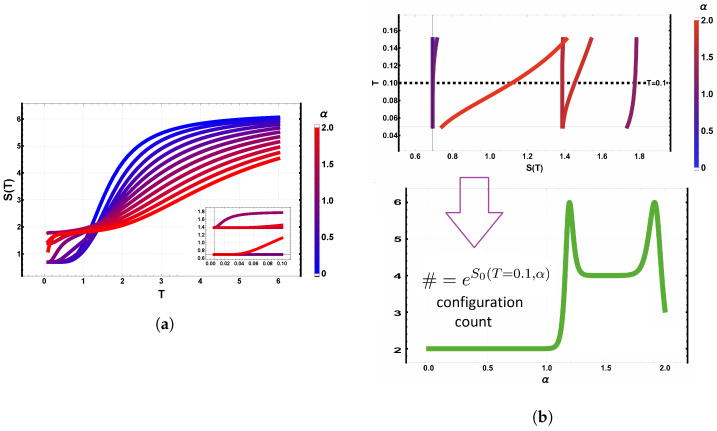
Entropy and ground-state structure for the Ising model on a 3×3 lattice as a function of α=D/J. (**a**) Entropy S(T) showing residual entropy plateaus reflecting changes in ground-state multiplicity. (**b**) Ground-state multiplicity versus α, exhibiting plateaus (twofold, fourfold, sixfold) that match the low-temperature entropy behavior. The inset shows the lowest energy levels whose crossings determine the multiplicity.

Figure 9a,b provide entropy-based and microscopic insight into the same dependence on α. Figure 9a displays the entropy S(T) for all values of α. As T→0, the residual entropy plateaus reflect the ground-state multiplicity: near the edges of the explored interval, $S_{0}=\ln2$, while around α≈1 the plateau increases, consistent with a higher multiplicity such as a fourfold ground state. At high temperature, all curves converge to a disordered state with a resistance to disorder as the dipolar term increases.

Figure 9b confirms this interpretation by showing explicitly the ground-state multiplicity as a function of α. Distinct plateaus (e.g., two, four, or six configurations) appear in separate α intervals, and the inset highlights the evolution of the lowest-energy levels with α. Level crossings between nearly degenerate states account for the observed jumps in the multiplicity and for the entropy plateaus shown in panel (a).

### 3.4. Q-Clock Models: Dependence on the Dipolar Ratio

**Figure 10:**
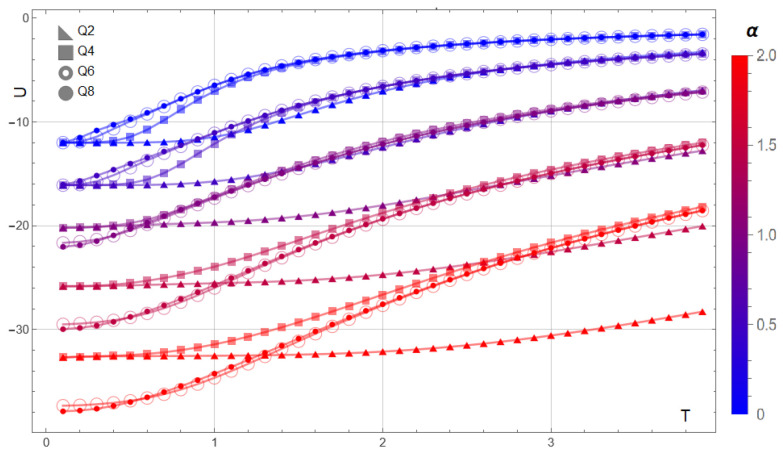
Internal energy U(T) for a 3×3 lattice and clock symmetries Q=2,4,6,8 at selected dipolar ratios α=D/J∈{0,0.5,1,1.5,2} (color bar from blue to red). Increasing α lowers the low-temperature energy and produces a systematic split between Q={2,4} and Q={6,8}, reflecting the stronger influence of long-range couplings in clocks with greater angular freedom. At high temperature the curves approach their interaction-dependent saturation; for α≃1.5 the Q≥4 energies cluster close to the Ising case at α=0.

Figure 10 summarizes the internal energy for Q=2,4,6,8 on a 3×3 lattice (no magnetic field) when the dipolar ratio is varied as α=D/J∈{0,0.5,1,1.5,2}. At low temperatures, increasing α lowers the energy for all *Q*, and a clear separation emerges between two groups: Q={2,4} and Q={6,8}. This split is consistent with the larger angular freedom of the higher-*Q* clocks, which enhances the impact of long-range interactions on the low-energy manifold. At high temperatures, each family approaches its interaction-dependent saturation, and for sufficiently strong dipolar coupling (e.g., α≈1.5) the Q≥4 curves cluster near the Ising baseline at α=0, indicating a partial compensation between exchange and dipolar contributions.

**Figure 11:**
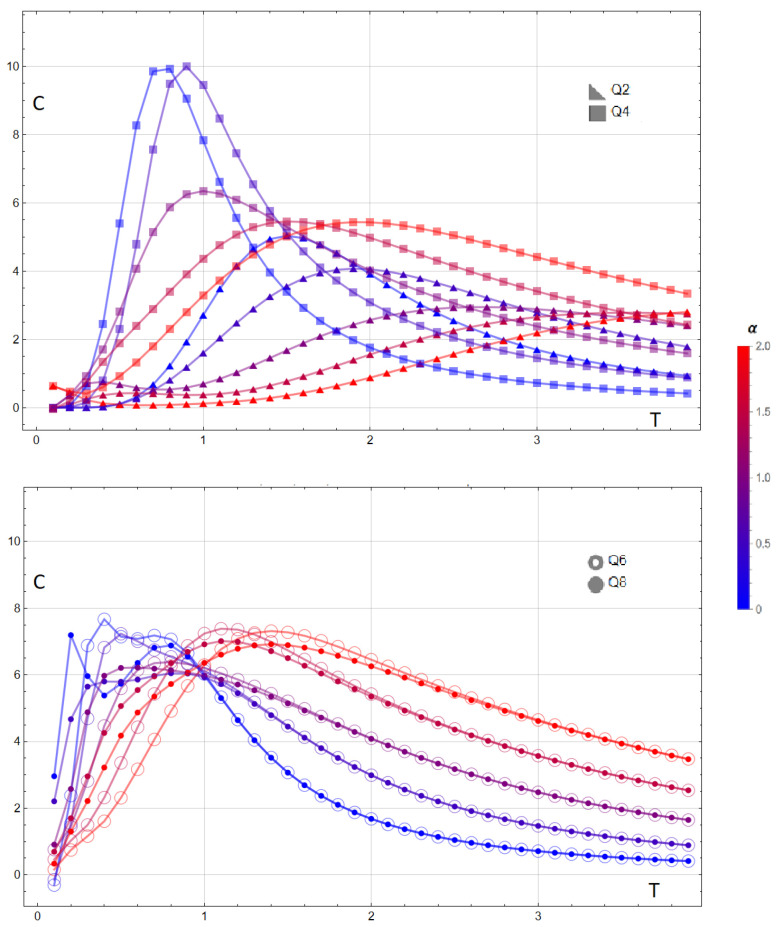
Specific heat C(T,α) for a 3×3 lattice and clock symmetries Q=2,4 (**top**) and Q=6,8 (**bottom**) at selected dipolar ratios α=D/J∈{0.0,0.5,1.0,1.5,2.0} (color bar from blue to red). For Q=2,4, the main maximum shifts to higher *T* and broadens with increasing α; a low-temperature shoulder appears for Q=2 at intermediate α. For Q=6,8, the double-peak structure present at small α is gradually suppressed, yielding a single broad maximum at larger α and nearly indistinguishable high-*T* tails.

The specific heat reveals a systematic evolution with the dipolar ratio α=D/J across clock symmetries. For Q=2 and Q=4 (top panel of Figure 11), increasing α shifts the main maximum of C(T,α) to higher temperatures and broadens the curve; in the Ising case (Q=2) a low-*T* shoulder emerges at intermediate ratios, whereas for Q=4 the response remains single-peaked with a reduced height. For Q=6 and Q=8 (bottom panel), the double-peak structure evident at small α is progressively suppressed and merges into a single broad maximum as α grows, while the high-*T* tails become nearly *Q*-indistinguishable. These trends indicate that long-range dipolar couplings smoothen exchange-driven anomalies and push the dominant energy fluctuations to higher temperatures.

**Figure 12:**
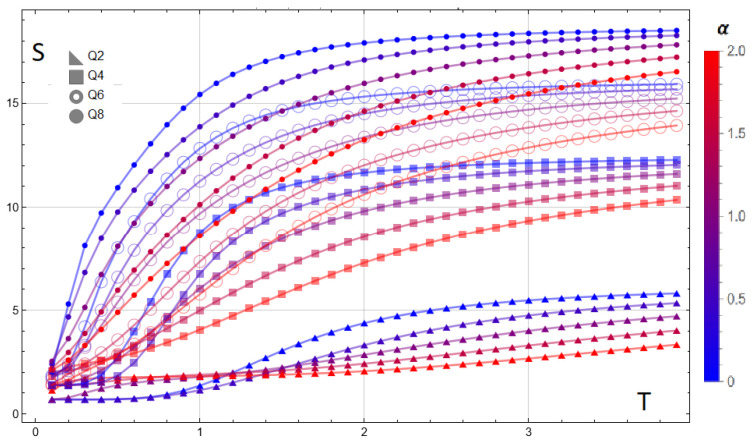
Entropy S(T) for a 3×3 lattice and clock symmetries Q=2,4,6,8 at selected dipolar ratios α=D/J∈{0,0.5,1,1.5,2} (color bar from blue to red). For fixed α, S/N increases with *Q* and tends to the high-*T* limit lnQ. Larger α lowers the curves at intermediate and high temperatures for Q≥4, evidencing the ordering effect of long-range interactions. At low temperatures, α-dependent plateaus reveal changes in ground-state multiplicity (residual entropy $S_{0}$), consistent with α-driven configuration phases.

**Figure 13:**
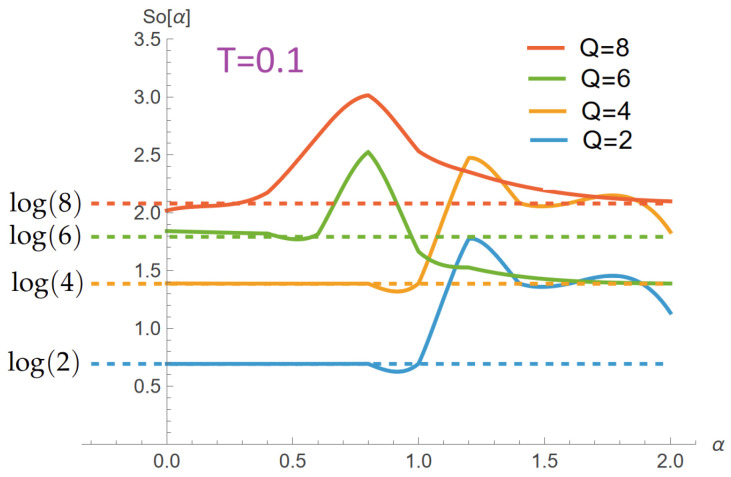
Residual entropy $S_{0}\left(\alpha ,Q\right)$ at T=0.1 for a 3×3 lattice and clock symmetries Q=2,4,6,8 as a function of the dipolar ratio α=D/J. Dashed lines mark the pure-exchange baselines lnQ. Deviations from these baselines (peaks/steps) reflect α-induced changes in ground-state multiplicity due to level crossings, with more pronounced effects for larger *Q*.

The entropy exhibits the expected high-temperature ordering with respect to clock symmetry (Figure 12): for fixed α, S/N increases monotonically with *Q* and approaches its asymptotic limit lnQ. Increasing the dipolar ratio α systematically lowers S(T) at intermediate and high temperatures for Q≥4, indicating that long-range couplings reduce the number of effectively accessible configurations. At low temperatures, the residual entropy $S_{0}\left(\alpha ,Q\right)$ reflects the α-dependent ground-state multiplicity: plateaus change with α, signaling zero-temperature configuration phases whose degeneracies differ from the pure-exchange expectation. A complementary view is provided by the residual entropy $S_{0}\left(\alpha ,Q\right)$ extracted at low temperature (here evaluated at T=0.1): as shown in Figure 13, the dashed baselines at lnQ represent the pure-exchange expectation, while the solid curves display α-dependent enhancements and depressions that signal reorganizations of the ground-state manifold. Peaks and step-like features indicate accidental degeneracies created by level crossings as the dipolar term competes with exchange; the effect is broader and more pronounced for larger *Q* (greater angular freedom), whereas for small and large α the curves tend back toward their lnQ baselines. These trends corroborate the plateaus observed in S(T) at T→0 and quantify how long-range couplings reshape the zero-temperature configuration space.

### 3.5. Approach to Magnetization

**Figure 14:**
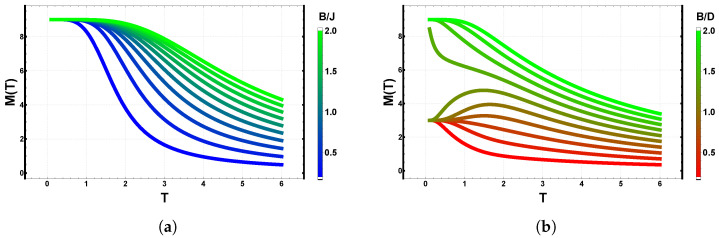
Ising magnetization M(T) for exchange–Zeeman and dipolar–Zeeman couplings on a 3×3 lattice (Q=2, L=3). (**a**) Exchange interaction with different values of the external magnetic field *B*. (**b**) Dipolar interaction with different values of the external magnetic field *B*. Color bars indicate the relative field strength in each case.

In the dominant energy regime, i.e., for temperatures below the Onsager critical temperature, an external magnetic field promotes a spontaneous global spin alignment, leading to a ferromagnetic configuration. While the exchange interaction drives local spin alignment between neighboring sites, achieving a fully ordered state requires a larger energetic contribution (see Figure 4 and Figure 5). In contrast, the texture that minimizes the magnetostatic energy in the dipolar case does not favor global alignment; instead, it induces magnetic domains with radial anisotropy, producing two-dimensional patterns similar to sources or sinks. This competition effectively shifts the critical temperature $T_{C}$ toward higher values. In simple terms, the exchange term reinforces the Zeeman contribution, whereas the dipolar interaction competes with it, defining the dominant texture of the system. To visualize these effects, Figure 14 presents the temperature-dependent magnetization M(T) obtained from the free energy of the Ising model for L=3 and Q=2, under two distinct energetic scenarios:

(8) $$\begin{array}{cc} E & =E^{\left(J\right)}-B\;\sum_{i}\cos\theta_{i},\\ E & =E^{\left(D\right)}-B\;\sum_{i}\cos\theta_{i}. \end{array}$$

where *B* denotes the external magnetic field, expressed in units of the exchange or dipolar coupling strength, and varied within the range $B_{\min}=0.2$ to $B_{\max}=2.0$.

The figure shows that the Zeeman term enhances spin alignment at low temperatures across the entire field range. For the exchange–Zeeman case (blue-green curves), the system saturates rapidly even at weak magnetic fields, reaching maximal magnetization at low temperature. In contrast, for the dipolar–Zeeman case (red-green curves), saturation is not achieved within the same field interval. Above a characteristic threshold $B^{*}$, the competition between dipolar anisotropy and the external field prevents global alignment, leading instead to partially ordered or domain-like spin configurations [15,16,37].

### 3.6. Low-Temperature Shoulder and Secondary Peak in C(T,α)

**Figure 15:**
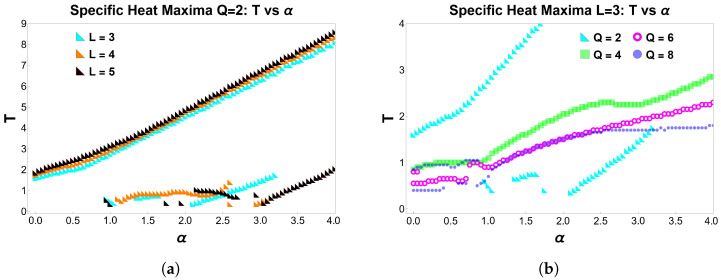
Peak temperatures of the specific heat C(T,α) as a function of the dipolar ratio α=D/J. Each symbol marks a local maximum of C(T,α) obtained from the full temperature dependence at fixed α. (**a**) Ising case (Q=2) for L=3,4,5. Two points with the same color at a given α indicate the coexistence of primary and secondary maxima. Intervals without points indicate that no local maximum is present in that temperature window. (**b**) Clock models for a 3×3 lattice and Q=2,4,6,8, showing the corresponding symmetry dependence of the peak temperatures.

The evolution of the specific heat for dipolar Ising and clock clusters reveals nontrivial finite-size structure beyond the main ferro–paramagnetic peak. In particular, the presence of low-temperature shoulders and secondary maxima reflects how competing interactions reshape the low-energy spectrum as the dipolar ratio α=D/J is varied. These features are summarized in Figure 15, which condenses the temperature locations of all local maxima of C(T,α) as functions of α.

Figure 15a focuses on the Ising case (Q=2) for lattice sizes L=3,4,5. Each symbol marks a local maximum of C(T,α) identified from the full temperature dependence at fixed α. The main ferro–paramagnetic peak is present for all α and shifts monotonically to higher temperatures, showing only a weak dependence on *L*, consistent with bulk-like excitations controlling this crossover. In contrast, the low-temperature peak appears only in restricted intervals of α and is strongly size dependent.

Following a single color-coded branch in Figure 15a for Q=2, one observes that for small α only a single maximum is present, as expected for the standard Ising model dominated by short-range exchange interactions. Beyond a critical value of α, a second maximum emerges at lower temperatures, leading to the coexistence of a primary and a secondary peak. This additional low-temperature maximum is not anticipated in the pure Ising limit and signals a dipolar-induced reorganization of the low-energy spectrum.

Importantly, the absence of points in certain α intervals does not indicate missing data but rather signals that no local maximum exists in that temperature range. In those intervals, C(T,α) instead develops a local minimum (or vanishes within numerical resolution), implying that the system crosses over smoothly between low-lying energy manifolds without accumulating spectral weight at finite temperature. These “white regions” in the peak map correspond to values of α where the specific heat approaches zero as T→0, before re-emerging at finite temperature as α is further increased. As will be discussed in detail below, these gaps are closely connected to changes in the structure and degeneracy of the ground state induced by dipolar interactions.

We emphasize that the branch-following discussion above refers exclusively to Figure 15a, where the lattice size *L* is varied for the Ising case (Q=2). Figure 15b, by contrast, fixes the lattice size to L=3 and illustrates how the thermal response evolves as a function of the clock symmetry *Q*.

Figure 15b shows the evolution of the specific-heat maxima as a function of the dipolar ratio α for a 3×3 lattice and several values of *Q*. The dominant peak shifts monotonically to higher temperatures as α increases for all *Q*, reflecting the growing energetic weight of long-range dipolar interactions. In addition, a secondary low-temperature maximum appears over a broad interval around α∼1. The occurrence of two points with the same color at a fixed α directly reflects this double-peak structure of C(T,α).

For the clock models, the apparent smoothing and partial overlap of the curves in Figure 15b originate from the increased density of low-energy states and from finite-temperature broadening inherent to the small cluster size. As *Q* increases, the discrete angular structure becomes finer, and the low-energy spectrum evolves more continuously with α, leading to broader and less sharply separated thermal response scales.

For Q>4, this behavior is consistent with a finite-size crossover regime of the dipolar clock model, where competing low-energy configurations give rise to multiple thermal response scales in small systems. In the Ising case (Q=2), the secondary peak remains but appears shifted and less pronounced, indicating that it arises from finite-size rearrangements of the discrete energy spectrum rather than from a genuine collective crossover or thermodynamic transition.

For the clock models at fixed L=3, as shown in Figure 15b, two distinct α intervals can be identified in which a secondary low-temperature peak emerges, particularly for Q≥4. These regions correspond to regimes where several low-energy configurations become nearly degenerate, enhancing thermal fluctuations at temperatures well below the main peak of C(T,α). Outside these intervals, the low-temperature structure disappears, again leading to gaps in the peak map that reflect smooth spectral reorganizations rather than sharp thermodynamic features.

Overall, Figure 15 should not be interpreted as a phase diagram, but rather as a compact map of the characteristic thermal response scales encoded in C(T,α) for finite clusters. The presence, absence, or multiplicity of peaks reflects how finite-size level crossings, boundary effects, and interaction competition organize the low-energy spectrum.

In the following discussions, we complement this analysis with contour plots of C(T,α) and a detailed discussion of the ground-state structure, which together clarify the microscopic origin of these discontinuities.

**Figure 16:**
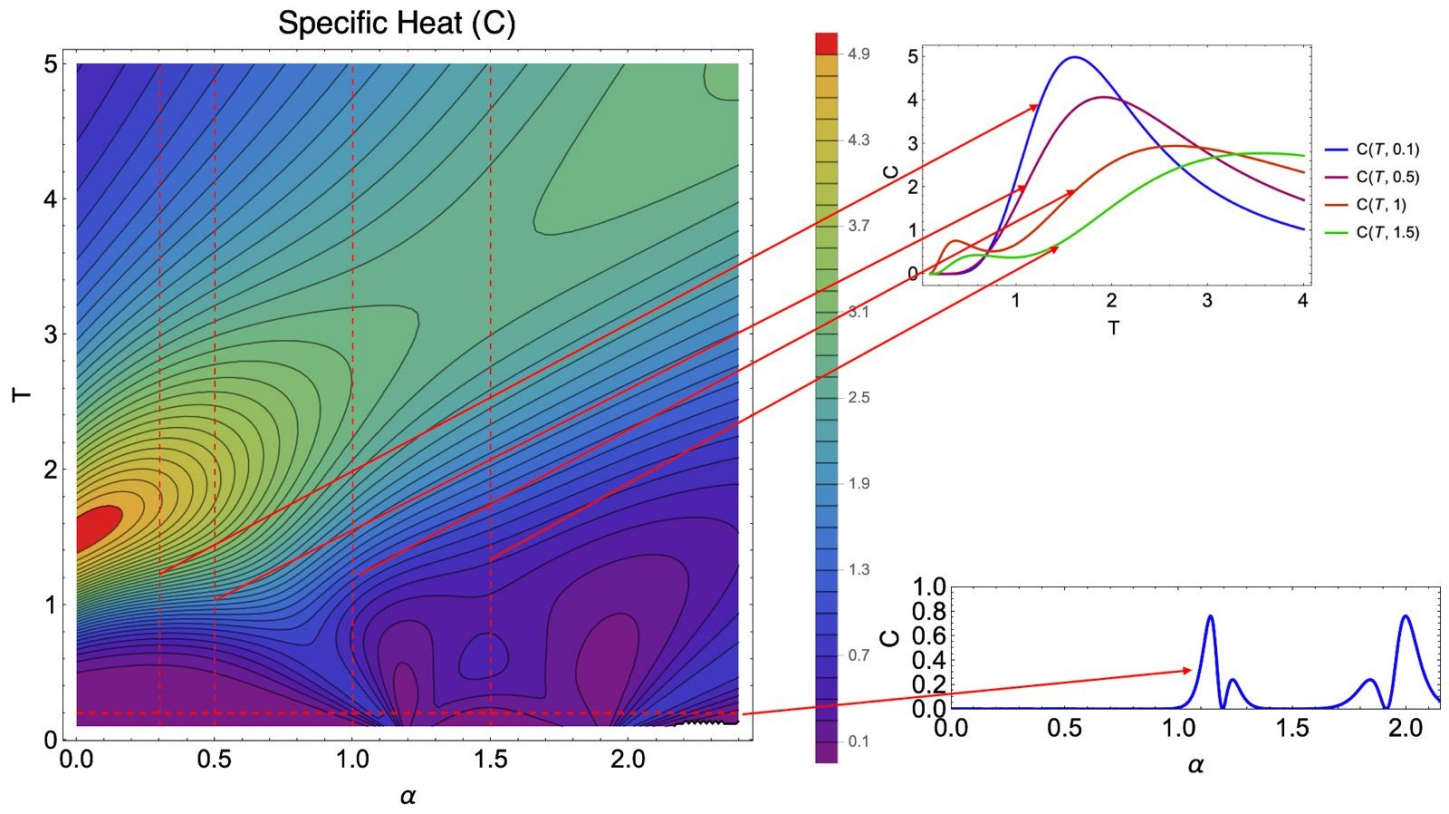
(Color online) Specific heat C(T,α) as a function of temperature *T* and the interaction ratio α in zero external field. The contour plot highlights low-temperature minima associated with ground-state level crossings, while the vertical cuts (right panels) show thermal activation peaks for selected values of α. The lower inset displays C(T→0,α), emphasizing the sequence of zeros at the critical points of the model.

Figure 16 synthesizes the two complementary mechanisms that govern the specific heat landscape of the model in the absence of an external magnetic field. In the low-temperature limit, the contour plot reveals a sequence of sharp minima along horizontal cuts at T≈0. These zeros of the specific heat C(T,α) occur at well-defined values of α and correspond to critical points where the ground state changes discontinuously due to level crossings in the energy spectrum. Because thermal excitations are suppressed at these isolated points, the specific heat vanishes as T→0, producing the pronounced dips highlighted in the lower inset.

In addition to these ground-state transitions, the figure also exhibits clear signatures of thermal crossovers at finite temperature. For intermediate and large values of α—where the long-range dipolar interaction becomes comparable to or dominates the exchange term—the system develops a nontrivial temperature dependence. As illustrated by the vertical cuts (upper right panel), C(T,α) shows pronounced maxima arising from the thermal population of excited configurations with competing spin arrangements.

Since the dipolar interaction favors antiferromagnetic alignment, whereas the exchange interaction stabilizes a ferromagnetic arrangement, their competition gives rise to a progressive reorganization of the dominant spin correlations as temperature increases, before the system ultimately crosses over to the paramagnetic regime. This interplay accounts for the broad high-temperature peaks observed at large α, including those persisting in the Ising limit. Overall, the C(T,α) map provides a unified view of the ground-state transitions and finite-temperature crossovers shaping the thermodynamic behavior of the system.

**Figure 17:**
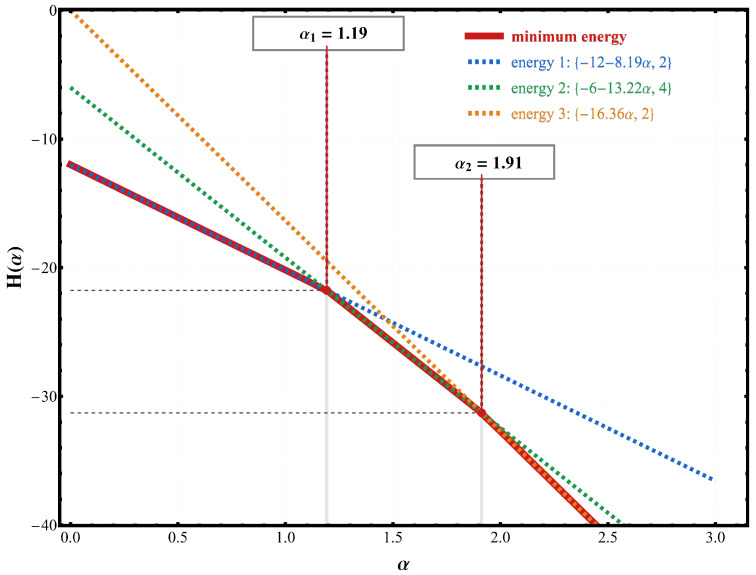
(Color online) Energies of the lowest competing configurations as a function of the interaction ratio α. The red solid curve marks the minimum energy branch, while the dashed lines indicate the excited configurations that successively become the ground state. The crossings at $\alpha_{1}$ and $\alpha_{2}$ correspond to the critical values where the ground state changes discontinuously, in agreement with the low-temperature structure observed in the specific-heat map.

Figure 17 provides a direct and transparent confirmation of the critical values of α identified from the specific heat analysis. The dashed branches represent the energies of the lowest competing spin configurations, while the red curve tracks the lowest among them as a function of α. The two crossings at $\alpha_{1}≃1.19$ and $\alpha_{2}≃1.91$ mark the points where the ground state changes discontinuously. These are precisely the same values at which the specific heat exhibits zeros in the limit T→0 in Figure 16, confirming that the low-temperature anomalies originate from level crossings in the energy spectrum.

An essential feature of these crossings is that the level degeneracy is not symmetric around the critical points. As a consequence, the corresponding Schottky anomaly does not produce a symmetric peak in the specific heat on both sides of the transition. Before the first crossing, the energy gap and its degeneracy give rise to a well-defined activated peak. However, beyond the critical value, the ordering of the levels is reversed, and the accessible low-lying excitations change, modifying both the position and amplitude of the Schottky contribution. This explains the asymmetric shape of the low-temperature peaks and the distinct behavior observed before and after each critical point α.

**Figure 18:**
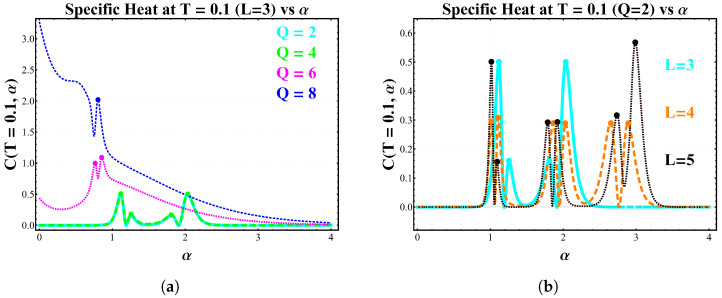
(Color online) Low-temperature specific-heat peaks at T=0.1 as a function of the dipolar ratio α=D/J. (**a**) 3×3 lattice for several *Q* in the *Q*-state clock model: sharp peaks for Q=2,4 signal ground-state crossings, while larger *Q* values show only smooth thermal features. At this temperature, the curves for Q=2 and Q=4 are nearly superimposed over a wide range of α, reflecting the similarity of their low-energy excitation structure at very low *T*. (**b**) Ising case (Q=2) for lattice sizes L=3,4,5: odd lattices display two critical points with asymmetric Schottky-like anomalies, whereas the even lattice (L=4) exhibits three critical points accompanied by nearly symmetric peaks.

Figure 18a shows how the low-temperature specific-heat structure evolves as the number of local spin states *Q* increases for a 3×3 lattice. For Q=2 and Q=4, the curves exhibit a sequence of sharply defined peaks located at the same values of α identified in Figure 16 and Figure 17. These peaks originate from ground-state crossings, and their positions coincide with the critical values at which the lowest-energy configuration changes discontinuously. In contrast, for Q=6 and Q=8 the behavior changes qualitatively: the sharp low-temperature features disappear, and the specific heat displays only broad thermally activated maxima. This indicates that, for larger *Q*, the energy landscape no longer produces ground-state crossings within the relevant range of α. The increased number of accessible configurations smooths out the level structure, removing the abrupt degeneracy changes that generate zeros of the specific heat as T→0. Thus, only the Q=2 and Q=4 cases retain the critical behavior associated with level crossings, whereas systems with Q≥6 exhibit purely thermal signatures.

Figure 18b highlights a striking dependence of the low-temperature specific heat on the parity of the lattice size *L* in the Ising case (Q=2). For the odd lattices L=3 and L=5, the curves display exactly two critical values of α, consistent with the two ground-state crossings identified in the energy analysis. Immediately after each zero of the specific heat, a Schottky-type anomaly develops; however, these peaks are strongly asymmetric. This lack of symmetry reflects the fact that the degeneracies of the competing energy levels are not equivalent on both sides of the crossing. Once the ground state changes, the multiplicities of the first excited configurations differ, producing an imbalance in the thermal population that manifests as a distorted Schottky profile. The behavior for the even lattice L=4 is markedly different. In this case, three critical points appear within the same interval of α, leading to three zeros in the low-temperature limit of the specific heat. Moreover, the associated Schottky peaks are significantly more symmetric. Such symmetry indicates that the levels involved in each crossing possess comparable degeneracies, so the activated contribution rises and falls in a nearly balanced manner around the critical point.

Altogether, panels (a) and (b) of Figure 18 show that the symmetry (or asymmetry) of the Schottky anomaly acts as a direct fingerprint of the degeneracy structure of the low-lying spectrum. Odd lattices, with unbalanced multiplicities across the crossings, yield asymmetric peaks, while even lattices tend to produce more symmetric anomalies due to the pairing of degeneracies. This establishes a clear connection between lattice parity, ground-state structure, and the thermodynamic signatures of the model (Table 2).

To make the degeneracy–caloric correspondence fully explicit, we summarize in Table 3 representative ground-state multiplicities across the α-driven crossings for the Ising case (Q=2) and lattice sizes L=3,4,5. This compact bookkeeping highlights how changes in ground-state degeneracy correlate directly with the appearance of zero-temperature specific-heat zeros and with the symmetry or asymmetry of the associated Schottky anomalies.

These values are sufficient to rationalize the parity-dependent caloric response discussed above and confirm that the observed features originate from discrete spectral reorganizations of the low-energy manifold rather than from collective critical phenomena.

## 4. Experimental Relevance and Feasibility

Although the present work focuses on exact finite-cluster thermodynamics, the control parameter explored throughout the manuscript, namely the ratio α=D/J between dipolar and exchange interactions, is directly connected to microscopic energy scales that can be realized in a variety of experimental platforms.

In bulk van der Waals magnetic materials, nearest-neighbor exchange couplings typically lie in the range J∼1–10 meV, while dipolar interactions are much weaker, $D∼10^{-3}$–$10^{-2}$ meV, leading to α≪1. In this regime, dipolar effects act primarily as a weak perturbation to exchange-dominated magnetism. However, this hierarchy can be substantially modified in nanostructured or weakly coupled van der Waals systems, where reduced coordination, spatial confinement, or engineered geometries suppress effective exchange pathways and enhance the relative importance of dipolar anisotropy.

Paramagnetic salts constitute a particularly relevant realization of dilute magnetic systems with localized moments. In these materials, exchange interactions are often extremely weak ($J∼10^{-3}$–$10^{-2}$ meV), while dipolar couplings remain finite, yielding effective ratios α∼0.1–1. This places paramagnetic salts squarely within the parameter regime explored in the present finite-cluster analysis, especially in the low-temperature limit where discrete level structures dominate thermodynamic responses.

Artificial spin-ice arrays and magnetic nanoisland clusters represent an even more direct realization of dipolar-dominated physics. In these systems, the interaction energy scale is set almost entirely by dipolar coupling, with characteristic energies ranging from ∼$10^{-2}$ to 1 meV depending on island size, shape, and spacing, while exchange interactions are negligible or fully design-dependent. As a result, effective values α≳1 are naturally achieved, and clusters with linear dimensions comparable to those studied here (L=3–5) are routinely fabricated and experimentally characterized.

These order-of-magnitude estimates are summarized in Table 4, which demonstrates that the range of α investigated in this work (α∼0.1–2) spans experimentally accessible regimes across several relevant platforms, from weakly dipolar exchange-dominated materials to genuinely dipolar mesoscopic systems.

Regarding temperature scales, the low-energy features identified in our analysis correspond to temperatures ranging from sub-kelvin to a few tens of kelvin, depending on the microscopic energy scale. Such temperature windows are well within reach of modern cryogenic techniques. Importantly, our focus on exact finite-size thermodynamics is particularly appropriate for these platforms, where system sizes are intrinsically small and boundary effects are not a finite-size artifact but an essential physical ingredient.

## 5. Conclusions

In this work, we have carried out a detailed and fully controlled thermodynamic analysis of small dipolar *Q*-state clock lattices, with particular emphasis on the 3×3 geometry, where exact enumeration allows the complete microscopic spectrum and its degeneracy structure to be resolved without approximation. This approach demonstrates that, even in the Ising limit (Q=2), the inclusion of long-range dipolar interactions is sufficient to generate nontrivial low-temperature caloric features. In particular, additional low-temperature peaks appear in the specific heat at well-defined values of the interaction ratio α=D/J, signaling a reorganization of the low-energy manifold that is absent in the pure exchange model.

A central result of this study is the establishment of an exact, degeneracy-resolved correspondence between microscopic spectral rearrangements and macroscopic thermodynamic signatures. We identify sharp critical values of α that manifest as exact zeros of the specific heat in the limit T→0. These minima coincide with discontinuous changes in the ground state driven by level crossings in the energy spectrum, providing an unambiguous thermodynamic fingerprint of dipolar-driven ground-state reorganizations. Contour maps of C(T,α), together with an explicit tracking of the lowest energy branches, confirm that each zero of C(T→0,α) originates from such a crossing.

By comparing different lattice sizes, we have shown that the shape of the Schottky-like anomaly following each critical point encodes detailed information about the degeneracy structure of the competing low-lying levels. For odd lattices (L=3 and L=5), the post-critical peaks are strongly asymmetric, reflecting unbalanced multiplicities across the crossings. In contrast, the even lattice (L=4) exhibits three critical values of α accompanied by nearly symmetric Schottky anomalies, indicating that the levels exchanging stability possess comparable degeneracies.

These results identify lattice parity as a key organizing principle governing the symmetry or asymmetry of low-temperature caloric responses in finite dipolar clusters.

For larger clock symmetries, the phenomenology changes qualitatively. While systems with Q=2 and Q=4 retain sharp critical values of α associated with ground-state crossings, for Q≥6 the energy landscape becomes sufficiently smooth that such crossings are suppressed within the explored parameter range. As a result, the low-temperature anomalies disappear and the specific heat displays only broad, purely thermal maxima. The absence of a genuine Berezinskii–Kosterlitz–Thouless transition in the finite clusters studied here is not a consequence of dipolar interactions alone, but rather reflects the combined effect of finite system size and the underlying discrete clock symmetry, which preclude the development of true topological order. Nevertheless, the evolution observed for Q≥6 bears a qualitative resemblance to the crossover behavior associated with vortex–antivortex physics in the two-dimensional XY model, in the sense that increasing angular freedom progressively smooths degeneracy-driven features.

Taken together, our results demonstrate that finite dipolar clock clusters constitute controlled benchmark systems in which long-range anisotropy, ground-state degeneracy, and finite-size geometry act on equal footing. The combined analysis of specific-heat extrema, residual entropies, and degeneracy patterns provides a coherent microscopic interpretation of the thermal anomalies induced by dipolar interactions. These findings are directly relevant to mesoscopic platforms such as artificial spin-ice arrays, dipolar-coupled nanoislands, paramagnetic salts, and confined van der Waals nanomagnets, where finite size and boundary effects are intrinsic rather than a nuisance.

They also provide a firm foundation for future extensions to larger clusters and to dynamical or field-driven regimes, where the same competition between exchange and dipolar couplings is expected to generate similarly intricate thermal landscapes.

## Figures and Tables

**Table 1:** Number of spin configurations as a function of lattice size *L* and number of states *Q*. Values shown in bold indicate the parameter sets that are explicitly calculated and analyzed throughout this work.

*L*	Q=2	Q=4	Q=6	Q=8
**3**	$5\times 10^{2}$	$3\times 10^{5}$	$1\times 10^{7}$	$1\times 10^{8}$
**4**	$7\times 10^{4}$	$4\times 10^{9}$	$3\times 10^{12}$	$3\times 10^{14}$
**5**	$3\times 10^{7}$	$1\times 10^{15}$	$3\times 10^{19}$	$4\times 10^{22}$
6	$7\times 10^{10}$	$5\times 10^{21}$	$1\times 10^{28}$	$3\times 10^{32}$
7	$6\times 10^{14}$	$3\times 10^{29}$	$1\times 10^{38}$	$2\times 10^{44}$

**Table 2:** Summary of low-temperature specific-heat features for the Ising case (Q=2) as a function of lattice size *L*. The number of critical dipolar ratios $N_{\mathrm{crit}}$ corresponds to the zeros of C(T→0,α), while the Schottky-peak shape and degeneracy pattern reflect the structure of the low-lying spectrum discussed in Figure 17 and Figure 18.

*L*	*Q*	$N_{\mathrm{crit}}$	Schottky-Peak Shape	Degeneracy Pattern
3	2	2	Strongly asymmetric	Unbalanced low-lying degeneracies
4	2	3	Nearly symmetric	Approximately paired degeneracies
5	2	2	Asymmetric	Unbalanced degeneracies (odd parity)

**Table 3:** Representative ground-state (GS) degeneracies for the Ising case (Q=2) as a function of the dipolar ratio α=D/J. Here $\alpha_{1},\alpha_{2},\alpha_{3}$ denote the critical values at which ground-state crossings occur.

*L*	α Regime	GS Character	$g_{\mathrm{GS}}$
3	$\alpha <\alpha_{1}$	ferromagnetic	2
3	$\alpha_{1}<\alpha <\alpha_{2}$	non-ferromagnetic manifold	4
3	$\alpha >\alpha_{2}$	distinct non-ferromagnetic pair	2
4	across $\alpha_{1},\alpha_{2},\alpha_{3}$	parity-even case	2 (throughout)
5	across $\alpha_{1},\alpha_{2}$	parity-odd case	2 (throughout)

**Table 4:** Order-of-magnitude estimates of the effective dipolar-to-exchange ratio α=D/J for representative physical platforms. The values are indicative and depend on microscopic details such as magnetic moment size, inter-site distance, lattice geometry, and confinement.

System	*J* (Typical Scale)	*D* (Typical Scale)	α=D/J
Bulk van der Waals magnets	1–10 meV	$10^{-3}$–$10^{-2}$ meV	$10^{-3}$–$10^{-2}$
vdW nanostructures/weakly coupled layers	0.1–1 meV	$10^{-3}$–$10^{-2}$ meV	$10^{-2}$–$10^{-1}$
Paramagnetic salts (dilute moments)	$10^{-3}$–$10^{-2}$ meV	$10^{-4}$–$10^{-3}$ meV	0.1–1
Artificial spin-ice arrays	negligible or design-dependent	$10^{-2}$–1 meV	≳1
Magnetic nanoisland clusters	weak/tunable	$10^{-2}$–1 meV	0.1–10

## Data Availability

The original contributions presented in this study are included in the article. Further inquiries can be directed to the corresponding authors.
